# Proceedings of the American Dental Association

**Published:** 1889-10

**Authors:** 


### Proceedings of the American Dental Association.

Special report of the Proceedings of the twenty-ninth Annual Session held at Saratoga, N. Y., August, 1889.

The opening session was held in the court chambers of the city hall, and was called to order at 11 A. M. by President C. R. Butler, of Cleveland, O. The president introduced Rev. R. D. Harper, of Philadelphia, who opened the convention with prayer. After prayer the roll was called. Reading of the minutes of the previous meeting, by vote, was dispensed with.

The Executive Committee made its report and submitted the programme for the approval of the association, which was, by vote, adopted.

Reports of the Publication Committee and Committee on Credentials were also made and approved.

The following amendment to the Constitution proposed last year, and under the rules having laid over for one year, was taken up and after considerable discussion laid on the table indefinitely: "Any permanent member of this association losing his membership in his local society from any cause, shall from that date cease to be a member of this Association."

The proposition to amend Section 5 of Article III. also failed to receive the required support.

Drs. Martindale, of Minneapolis, Bödecker and Carr, of New York, sent their resignations as members of the Association.

The Treasurer's report showed a balance of nearly $3,000.

After transacting the above business the president read his annual address.

Taking for his theme the influence of the lives of the former presidents of the American Dental Association upon the members of the dental profession, he paid a glowing tribute to the work and lives of Drs. Allport, Atkinson, Watt, Allen, McQuillen, Taft, Morgan, Keely, Rehwinkel, McKellops, and others, giving many instances of the personal help and encouragement which he had received from them.

The address was cordially received and referred to the Committee on Publication.

Before the opening of the first session of the convention there had been placed in each seat a circular calling attention to the merit and superiority of a certain copper amalgam. The circular contained what seemed to be a certificate of endorsement by six or eight very prominent members of the Association and some of them teachers in dental colleges. Dr. McKellops calling attention to the circular asked that these gentlemen be required to explain their connection with the matter, as such a proceeding was in direct violation of a resolution of the Association, adopted several years ago. The affair created considerable merriment, as the alleged signers one after the other arose and denied having ever given the manufacturers any certificate or authority to use his name in any such manner.

The meeting then adjourned until 7:30 P. M.

FIRST DAY—EVENING SESSION.

After reading the minutes of the morning session,

SECTION IV.—HISTOLOGY AND MICROSCOPY

Was called, and Dr. Frank Abbott, chairman, made a verbal report, stating that the section had only one paper to report and that was a paper on

THE FORMATION OF DENTAL ENAMEL, BY DR. FRANK ABBOTT, OF NEW YORK.

The object of Dr. Abbott's paper was to affirm the existence of a layer of small globular pearl-colored bodies on the surface of the enamel organ or ameloblasts which, instead of, as many suppose, secreting the inorganic material for the formation of the enamel, are organized cells which appropriate the material and become themselves built up as the enamel rods.

Dr. Abbott submitted drawings of preparations in his possession maintaining his position. These drawings were criticised by Dr. Sudduth, who was of the opinion that the drawings did not fairly represent the microscopic preparations, stating that one was likely to overdraw the things in which he was interested and fail to reproduce other very important structures.

Dr. W. H. Atkinson—I wish to express my unqualified approval of this paper and these drawings. I do not believe a photograph will as clearly illustrate conditions of growing tissues as a drawing, for the reason that in a photograph the lines are indistinct, and there is extraneous matter, which has become attached to the tissue in its preparation for mounting, this is reproduced in the photograph, making it misleading if not valueless. I wish we could have more men who would engage in this work and tell us what they see not only with their eyes and microscopes, but what they see with their mental eyes as well.

This subject was further discussed by Dr. W. X. Sudduth on Wednesday evening. The doctor illustrated the subject with the micro-lantern, exhibiting about fifty slides, beginning with the formation of the cells in embryonal tissue, and following the development of the tooth germ through all the various stages of its growth. These slides are made directly from the microscopic sections, and are not re-touched. The doctor claimed that they more accurately represented the conditions of growing tissues than the drawings of Dr. Heitzmann. Dr. Sudduth does not think there can be demonstrated a special globule or cell which is organized into enamel; thinks the enamel organ secretes the inorganic material and that it is deposited in the matrix of the tooth, or the organic portion.

SECOND DAY.—MORNING SESSION.

SECTION V.—MATERIA MEDICA AND THERAPEUTICS.

Dr. A. W. Harlan read a paper entitled

GERMICIDES AND DISINFECTANTS.

The author referred to the value of essential oils as disinfectants and antiseptics; and stated that their value depended on the vaporizable camphors which are deposited at a temperature of 94° F. when sealed in the cavity of a living or pulpless tooth. Has made experiments with oil of cassia and oil of cinnamon separately, and found that the oil of cassia is more potent in restraining and preventing the growth of microbes.

Dr. W. C. Barrett--We are too apt to multiply drugs. I am not able to comprehend any material difference between oil of cassia and oil of cinnamon; think we have other remedies that are equally good germicides.

Dr. W. H. Atkinson--I do not think we know when the microbes are destroyed. The oil of cassia is very much cheaper than the oil of cinnamon.

SECTION VI.--PHYSIOLOGY AND ETIOLOGY.

Dr. H. A. Smith, chairman, made a report continuing the subject of

IMPLANTATION, from the report made last year.

Dr. Younger reported that during the year he had made twenty-six operations, all of which were successful except three. All of the teeth Dr. Younger implanted were old and dry teeth.

Dr. McFadden reported thirteen cases with no failures.

Dr. Hugenschmidt reported six cases with two failures.

Dr. W. N. Morrison reported twelve cases with two failures. Nine of the twelve cases were old teeth and three were freshly extracted. The roots were filled with gutta-percha and gold and sterilized with bichloride of mercury. The teeth were held in place by a vulcanized cap for thirty days. The two failures were of the freshly extracted teeth.

Dr. Louis Ottofy reported thirty cases; of these twenty-two were successful, four were doubtful and four were failures.

Dr. Smith recommended implantation as soon after extraction as the gum takes on a granulated condition, in the same socket from which the root has been extracted, and does not hesitate to excise the root of a tooth to implant it.

Dr. Watkins--Have implanted four teeth. They were old and dry. They have been implanted thirteen months and appear to be successful.

Dr. W. P. Horton--I called on Dr. Younger at his office and he assured me of the success of implantation. He claimed that no more failures followed this operation than were likely to occur in as many operations of equal importance in other parts of the body.

Dr. J. S. Marshall--I do not think we ought to consider replacing an extracted root with a tooth, in the original socket, without allowing time for the absorption of the process to take place, as a case of implanting, but of transplanting.

Dr. C. N. Peirce--Every tooth I have implanted that was denuded of periosteum has failed, and I have only been successful when I have used teeth with the dried periosteum remaining intact. I implanted one tooth, a superior second bicuspid, where there was considerable absorption of the alveolar process. In drilling out the socket my drill penetrated the antrum. The tooth was implanted, but in a few days the patient returned, saying that she was troubled with a constant hemorrhage from the nasal cavity. No special treatment was resorted to, but the patient was assured that it would be all right. In a few days the hemorrhage ceased and the case is a success.

Dr. G. L. Curtis--I consider it essential to success that the root be scraped and polished clean of all organic structures. I have had no failures in several operations.

Dr. Smith, of Denver--Dr. Younger tells me that he is very particular in selecting the teeth he uses for implanting. He will not implant a tooth if any portion of its periosteum is destroyed. But at the same time he does not hesitate to excise the root if it is too long. If he does, he is particular about filling the root and capping the end with gold. He will not use a tooth from a scorbutic or scrofulous person. Dr. Younger is not particular as to matching the color of the adjoining teeth, claiming that the natural pigment of an implanted tooth will be absorbed and replaced by the pigments contained in the fluids of the body in which the tooth is implanted. Dr. Younger's success is attributable to the care he uses in the selection of his patients and the thorough and careful manner in which he does his work.

Dr. Barrett--From the discussion, we learn that one man thinks it very important that the periosteum of the tooth remain intact; another has had complete success in many cases and attributes it to the fact that he removed every particle of organic material from the root and completely sterilized the implanted tooth. From this we must conclude that no definite method of procedure has as yet been discovered that will insure success.

Dr. Peirce—I claim that we have learned something from this comparison of methods and results, and one is that a tooth can be as successfully implanted with its periosteum all removed as though it were intact.

Dr. M. L. Rhein—I do not think sufficient time has elapsed since this operation was first performed to permit any of these operations being pronounced successful.

Dr. L. Ottofy—There are no extracted teeth on which the pericementum is perfect. At first I was impressed with the idea that the roots of teeth to be implanted should not be mutilated or cut, but I have had as good success with those teeth that I have excised as any others. I do not believe that an implanted tooth will assume the color of the natural teeth of the jaw in which it is implanted. To obtain a desirable color, or to prevent the chipping of the enamel, I sometimes implant a root on which I have set a Logan crown. I do not hesitate to cut a root as much as half if necessary. I think it is important to thoroughly sterilize the tooth to be implanted in a 1 to 2,000 solution of bichloride of mercury, and just before implanting to dip it in a 1 to 500 solution of the bichloride.

Dr. H. A. Smith—The statistics of Dr. Younger refer only to the cases performed in his own office and not to those performed at clinics.

Dr. Eugene S. Talbot read a paper on

### CLASSIFICATION OF IRREGUTARITIES OF THE TEETH.

As a result of a careful examination of over two thousand cases of irregular teeth, he has adopted a system of classifying the various forms of irregularity into V-shaped and saddle-shaped arches, and these are further modified into semi-V-shape, semi-saddle-shape, or half V and half saddle shape. These results, he claims, are produced because of the fixed character of the first molar, which he calls the base of the arch, and the cuspids are the keystones. The cuspids, erupting after the other teeth, and because of their long roots, will, in taking their places in the arch, either crowd the bicuspids inside of the arch, producing the saddle-shaped variety, or crowd the incisors forward, producing the pointed or V-shaped variety. Dr. Talbot has prepared a set of models illustrating all these varieties of irregularities, which will be placed in the National Museum at Washington.

### DISCUSSION.

Dr. W. C. Barrett—I am very glad to learn that this subject is being studied with the idea of determining the causes which produce these irregularities, and that a systematic classification is being made. This will render the adaptation of appliances for correction a more simple matter.

Dr. Smith, of Denver—I hope Dr. Talbot will continue this work until we shall have more definite knowledge as to the causes which produce these irregularities, then we will be able to employ the necessary means for their prevention.

Dr. Curtis called attention to the wonderful regularity of the teeth in the lower jaw in the cases cited by Dr. Talbot.

Dr. Louis Ottofy stated that the Illinois State Dental Society had undertaken to make an examination of all the prehistoric crania in this country, for the purpose of ascertaining the extent to which they were affected by caries, abrasion, irregularity, etc. Several States, Kentucky, Georgia, and California, have contributed money to assist in the work. He read a communication from the Illinois Society to the American Dental Association requesting that a committee be appointed from the Association and the work of examination and tabulating the statistics be placed in charge of this committee.

Dr. Barrett called attention to the fact that several years ago he had made just such an examination of over two thousand skulls, and the result was published in the *Independent Practitioner.* He was in sympathy with this movement and hoped the association would take the matter in charge, as there was much to be learned from studying these prehistoric races.

A motion was passed appropriatiug $500 for the use of a committee of five, to be appointed by the president of the Association, to undertake this work in connection with a committee from the Illinois State Society.

SECOND DAY.—AFTERNOON SESSION.

Dr Allport offered the following resolution, which was unanimously adopted:

"WHEREAS, the National Association of Dental Faculties has adopted a resolution declaring that, beginning with the session of 1891–92, all colleges which are members of the National Association of Dental Faculties shall require three courses of study, of not less than five months each, taken in separate years, for graduation; therefore, be it

*Resolved*, That no delegate shall be received by the American Dental Association from any college which has not subscribed to this requirement."

After discussing the propriety of sending delegates to the International Dental Congress, to be held in Paris this year, it was decided to appoint no delegates.

SECTION VII.—ANATOMY, PATHOLOGY, AND SURGERY.

Dr. T. W. Brophy read a paper on

### SURGICAL LESIONS OF THE TERMINAL BRANCHES OF THE FIFTH PAIR OF NERVES.

This paper did not attempt a consideration of the lesions of the portions of the nerve distributed to the strictly dental organs, pulp and pericementum, but referred to the portion which is distributed to the tissues of the face, antrum, tongue, and gums. He related a case where by wearing a lower denture which covered the orifice of the mental foramen—which, in extensive absorption sometimes takes its place on top of the lower jaw—and caused severe neuralgia of the inferior dental nerve and afterward involved the inferior maxillary nerve. First operated by cutting through the inferior maxilla to the inferior dental nerve and removing a section of it; this failing to relieve the neuralgia, the inferior dental nerve was cut and removed entirely from the canal at the inferior dental foramen. The patient still complained of pain in the side of the tongue, showing that the inferior maxillary nerve was involved through the lingual. Exsection is only indicated when the application of remedial agents have failed to give relief. Local applications, injections, and nerve stretching should be employed before resorting to surgical operations.

DISCUSSION.

Dr. M. L. Rhein—I have used the chloride of methyl in the treatment of all forms of facial neuralgia with very good success. The rapid evaporation of the chloride of methyl produces a lower temperature, which produces a paralysis of the nervi nervorum.

Dr. Marshall—I have practiced evulsion, or tearing out the affected nerve as far back in the tissues towards the nerve origin as possible, with good success. Have cured a case of facial neuralgia by opening the infra orbital canal within the floor of the orbit, and taking hold of the nerve with a pair of forceps and winding the nerve upon the forceps until it tears loose from the main trunk back in the orbit, and then removing it.

The Secretary read a paper written by Dr. M. G. Jenison, of Minneapolis, entitled,

ORAL SURGERY AND WHO SHOULD PRACTICE IT.

The author called attention to the careless and often bungling treatment of diseased conditions of the oral cavity by physicians, and urged that it was within the province of the dentist to treat almost every lesion about the mouth, and urged upon the profession better preparation for, and more frequent practicing of, oral surgery, and treatment of diseased conditions about the mouth.

SECTION I.—PROSTHETIC DENTISTRY, CHEMISTRY, AND METALLURGY.

No papers were reported under this section.

Prof. Winder, of Baltimore, presented a piece of removable bridge-work, made by Dr. Spencer, of Virginia. The bridge was made to replace the second bicuspid and first molar in the inferior arch on the right side. The first bicuspid and second molar were fitted with collars, on the top of which was soldered a flat cap with a transverse dove-tail groove; caps were then swedged for the bicuspid and molar, and to the under side was soldered transversely, or from the labial to the lingual side, a solid dove-tail or tongue to fit accurately into the corresponding groove in the cap on the collars. The porcelain teeth are then backed and soldered to the caps. The collars are cemented in place on the natural teeth and the bridge is inserted by sliding the tongues into the grooves from the labial side. The grooves are broader at the labial than at the lingual side, and so are the tongues, so that when the bridge is in place the outer cusps of the upper teeth hold it firmly in place.

Dr. G. L. Curtis, of Syracuse, presented a removable bridge designed to replace the same teeth as the above, and somewhat similar in construction. In this bridge a gold crown is made for the molar and one for the bicuspid; to the posterior side of the bicuspid and the anterior side of the molar crowns are soldered projections, or arms, the free ends projecting into the space to be bridged and furnished with dove-tail heads. Thin platinum is then burnished around these arms and their heads to form a matrix, which is soldered to the backings of the porcelain teeth of the bridge. The crowns are set in place with cement, and the bridge is adjusted by pressing it from above downwards over the projecting arms, or dowels, the heads fitting accurately into the slots made by the platinum matrices.

Both of these bridges were nicely made, and are easily made and very practicable.

### SECTION II.—DENTAL EDUCATION, LITERATURE AND NOMENCLATURE.

Dr. W. H. Atkinson, Chairman of the Section, read a paper entitled,

#### SOME THOUGHTS ON DENTAL EDUCATION.

The paper deplored the necessity for the maintenance of the so-called post graduate schools. Argued for a more systematic presentation of all branches of dental science and art in the regular dental colleges. Thought the tendency was to secure larger classes, and that more thought was given to this than to improving the quality of the teaching, all of which was calculated to lower the standard of dental education. Hoped the time would soon come when more time was devoted to the course of college instruction, and personal hobbies would be thrown aside and that a broad, liberal and comprehensive course of study would be demanded and attainable. For practitioners he urged the establishment of dental unions, with a room fitted for holding clinics, where a day could be spent together at least once a month by the practitioners of the neighborhood. These unions could be made of incalcuable benefit to the members, and would be the means of preparing work for presentation at the larger societies and associations.

Dr. Louis Jack read a paper entitled

#### THE DESIRABILITY OF INDEPENDENT DENTAL JOURNALISM.

The paper advocated the idea of publishing the dental journals independent of dealers in dental supplies, claiming that a free and honest expression of one's convictions was only possible where the journal was not connected with a trade association or dependent on it for existence.

DISCUSSION.

Dr. Barrett—Dentistry aspires to a higher plane than the trades, and I believe that our literature ought not be embarrassed by placing it on a level with the unfair and often unscrupulous competitions incident to trade life. Have had experience in independent journalism, and while it was not financially profitable, the journal with which I was connected always paid its bills from the receipts of its patrons. But independent journalism means more than financial solvency or ability to pay its debts. A journal to be independent needs the support of the minds and pens of the profession as well as the subscription fees. Its editor needs to feel that he represents the best thought and highest attainment and has the support of the best men in the profession.

While I would not for one moment say a word, or entertain a suspicion, against the integrity of the house that for years has printed the transactions of this association, and have nothing to say in the way of criticism of the manner in which the work has been done, yet I wish the transactions of this meeting could be printed by the Association.

Dr. Crouse—When I remember the difficulties we used to have to overcome, in order that our transactions might be printed by the Association, especially the difficulty of securing the funds to pay for it, I am very glad to have even a dental dealer step in and relieve us of the trouble and expense, especially as the work is better done than it ever was before. We have a fair business understanding with our publisher, and I don't see where there is anything that is humiliating in this purely business arrangement.

Dr. Ottofy—Two-thirds of the practicing dentists of Illinois are inelligible to membership in this association, because they are not graduates. The graduates from the post graduate schools have organized an association with the same aim as this one, and it is hoped that much good work will be done among those who can not work here.

### Section III.—Operative Dentistry.

Dr. N. S. Hoff, Secretary of the Section, made the report, reviewing the work of the past year, and noting the departments in which there had been any special activity. The portion of the report that brought out the most discussion was the presentation of the results of some experiments performed by Dr. Joseph Head, of Philadelphia, to show the comparative value of cotton saturated with carbolized cosmoline, oxy-chloride of zinc, and gutta percha as materials for filling root canals.

In these experiments, first, three old or dry teeth with single straight roots were used. The pulp chambers were opened and the canals enlarged to admit of easy access and insure a perfect adaptation. The teeth were then warmed to the temperature of the body and one filled with gutta percha, one with oxy-chloride and the third with cotton saturated with carbolized cosmoline. The crown cavities were each filled with gold and gutta percha. After the teeth had been thus carefully filled, they were soaked for twenty-five days in a solution of red aniline ink; the teeth were then removed from the solution and the root canals exposed with their fillings. It was found that in the tooth filled with gutta percha the ink had not only found its way in around the filling but had penetrated the entire dentine of the root, and the same thing had taken place with the tooth filled with oxy-chloride of zinc. But the root that had been filled with carbolized cosmoline and cotton showed no trace of the ink stain, except immediately about the apical foramen; on the contrary, the tooth had a greasy translucent appearance, indicating that the cosmoline had penetrated the entire dentine.

The same experiments performed with freshly extracted teeth produced somewhat different results. In the teeth filled with gutta percha and oxy-chloride of zinc, the ink had penetrated around the root filling as before, but the red stain was found only in a small layer at the outer border of the dentine, next to the cementum, while the portion of the dentine next to the root canal showed only a slight trace of the color of the ink, showing that by a process of osmosis the coloring matter of the ink had passed through the fluid remaining in the dental tubuli and was deposited at the peripheral ends. The tooth that was filled with the cotton and carbolized cosmolin showed no trace of the ink stain, but the cosmoline had only penetrated the dentine for a limited space just around the pulp canal, showing that the fluids contained in the tubuli had dammed out the cosmoline. Dr. Head is of the opinion that if the fresh teeth were to remain in the ink solution for a longer time they would show a correspondingly increased amount of the coloring matter of the ink deposited in the dentine.

DISCUSSION.

Dr. Barrett—The difficulty in filling root canals is to perfectly seal the apical foramen. This is not only the case with teeth having small and tortuous canals, but I believe many roots have more than one opening through which the nerve and artery enter to the pulp. I have seen cases where there was not only an opening at the apical end of the root, but further up along the side will be found another opening, or perhaps two, hence the difficulty of enlarging the canals to gain access, and of introducing any filling material that will exclude extraneous fluids.

Dr. A. O. Hunt—I have a number of specimens in my possession showing several openings into the root canal at other points than the apex of the root. I account for it on the supposition that in the development of the root from the papilla a small artery or nerve from the dental follicle to the papilla has been surrounded by the deposition of the inorganic material around it, and when the tooth is completely formed it remains as a connection between the pulp and peridental membrane.

Dr. A. W. Harlan—A tooth that is saturated with cosmoline will so change its color as to be objectionable. Am satisfied that a root can be so filled with gutta percha that it will exclude all moisture. My method is to thoroughly dry the root with hot air blasts and then introduce eucalyptol, which is diffusible and in which gutta percha is soluble. After wiping out the excess, I pump in chloro-percha, and then take up on a heated instrument a cone of gutta percha (never heat the gutta percha) and force it into the root, allowing excess of chlora-percha to flow out.

Dr. H. J. McKellops—I am one of the first to advocate the use of chloro-percha for filling roots, and I still consider it good practice. I do not think any man can dress out the majority of root canals so that they can be filled with cotton or gold satisfactorily.

Dr. S. A. White--I have had trouble in filling roots with chloro-percha, because of forcing it through the apical foramen. I prefer to use the long staple cotton, saturated with chlora-percha, and have no difficulty in introducing it into most roots. My favorite method of filling the six front teeth is with a plug of hickory wood, cut around with a sharp knife a short distance from the end, so it will break off readily, then dipping it in chloro-percha and driving it into the root and breaking it off where cut. This closes the apical foramen, and the canal can then be filled with almost any material.

Dr. Barrett—For many years I used cotton saturated with chloro-percha, but found it unreliable and have now abandoned it for the gutta percha cones.

Dr. Ward—I presume one will have most success with the method he habitually practices, but I have been compelled to extract many teeth because of the peridental inflammation following the insertion of fillings of chloro-percha. I have had the best success with the oxy-chloride of zinc.

Dr. W. H. Morgan—I have been filling roots of teeth for over forty years. I enlarge the canals without exception, unless it is impossible to do so, which is seldom the case, removing as much of the dental tissue as possible, so that there will be less organic material to disintegrate and cause inflammation of the peridental membrane. I am particularly anxious that the whole tooth shall be rendered antiseptic and the surrounding tissues made healthy before filling. I stop the apical foramen with a small gold, tin or lead wire, and then fill the body of the canal with oxy-chloride of zinc and gold. I think oxy-chloride of zinc best because it will absorb the gases resulting from the decomposition of the remaining organic fluids and tissues. I don't believe any root was ever perfectly filled with chloro-percha.

Dr. James Truman—I do not think it is possible to perfectly fill the root of a tooth, because it is impossible with any of the present materials to fill the tubuli of the dentine. I have made some experiments with the idea of securing an antiseptic agent that can be forced into the dental tubuli. Chloride of zinc answers the purpose more satisfactorily than any agent I have been able to find, but its use requires care. My method is to permanently stop the apical foramen, and then introduce cotton saturated with chloride of zinc and leave for three or four days; the zinc coagulates the organic materials. I fill the root permanently with oxy-chloride of zinc.

Dr. McKellops—I want to say that chloro-percha root fillings do not shrink if they are properly made. Oxy-chloride of zinc makes a good root filling, so does gold. Thirty years ago Dr. F. H. Badger suggested filling roots with gold, and a tooth that I then filled with gold is doing well to-day.

Dr. J. N. Crouse--Dr. Harlan says coagulants are bad. Dr. Truman says they are good. I use both methods, and I get good results as often from one as the other. What we need is to exercise judgment in the selection of the proper treatment in each case, and not confine ourselves to any one special method of treatment.

Dr. J. D. Patterson--In small and tortuous canals I fill with chloro-percha. In making the chloro-percha I use the filling material instead of base plate. In roots having ready access I close the apical foramen with a small plug of gutta percha and fill the balance of the canal with oxy-chloride of zinc, using a small pledget of cotton to force the oxy-chloride into the root canal.

Dr. J. Taft--It is a matter of considerable importance for us to discriminate in the matter of the age of the patient for whom we are operating. In young teeth the apical foramen is likely to be large, and great care, not only in the selection of a proper material with which to fill but also in the manner of its introduction, is necessary. There is also more likely to be decomposition in young teeth than old ones. If proper care is exercised there is little danger forcing the filling material through the root. A small broach introduced into the canal carefully will stop at the shoulder in the canal at the beginning of the apical foramen; the broach can then be marked, and also the instrument with which the filling is introduced, and there need not then be any uncertainty about stopping at exactly the right place.

Dr. W. H. Dwinelle--I have been filling roots of teeth for fifty years. I fill with gold. I roll the gold into cones on a Swiss broach and carry to the end of the canal. I don't think it makes much difference what the material is with which the apical foramen is closed, so that it is perfectly sealed. Do not think wood is a good material.

Dr. W. W. Allport—When the pulp dies in a tooth it leaves the dental tubuli filled with dead and decomposing organic matter, and if the root canal is filled the gases pass out through the cementum, and cause irritation of the peridental membrane.

My practice is to close the apical foramen with oxy-chloride of zinc, and then fill the root canal with peroxide of hydrogen and allow it to remain for a few minutes. I then remove the peroxide and thoroughly dry the tooth with hot air and hot instruments, and then fill the root canal with oxy-chloride of zinc.

Dr. Sudduth—It is of no importance that the organic material in the tooth be coagulated, but it is necessary that the root be put in an antiseptic condition before it is filled. If the apical foramen is perfectly sealed, and the opening from the crown also, it does not make much difference what the root filling is. Micro-organisms will not develop in a tooth if the air is excluded. Micro-organisms are not found in healthy dentine. They are found in disorganized dentine, because it is the micro-organisms that break down the dentine. They attack the organic portion of the tooth and leave the inorganic, which is dissolved by the acids secreted or formed by the micro-organisms themselves. The odor sometimes found in a dead dry tooth, the apex and crown of which have both been perfectly sealed, may be due to the development of some class of micro-organisms.

Dr. Smith, of Denver--I do not like oxy-chloride of zinc for root fillings, because it disintegrates; gutta percha is not good, because it shrinks; wood is unprofessional, and is used only by the backwoodsmen of Indiana—Cravens objects.

Dr. F. Y. Clark—If you will fill the apex of a root for a short distance and then fill the crown and leave the tooth for some time and then open, you will find an odor coming from the putrefaction, produced by the air germs introduced during treatment. It is almost malpractice to enlarge a pulp canal. If the canal is thoroughly cleansed and disinfected with pure carbolic acid or creosote no decomposition will take place in twenty years.

Dr. Curtis read a paper written by Dr. Wilhelm Herbst, of Germany, entitled

FILLING TEETH WITH GLASS.

The paper described a method of making a glass inlay. Two kinds of glass are used, one the white glass of which lamp shades are made, and the other brown flint glass, such as chloroform bottles are made of. Each kind of glass is pulverized separately to a fine flour in a porcelain or agate mortar, in which no metal of any sort has been used. Wash the powdered glass with clear water until it is perfectly clean, dry and put in bottles for use. Cut out the cavity to be filled regular, with no undercuts, and be careful not to round off the edges. Take impression of the cavity with wax or compound and pour up the impression with gypsum and pulverized pumice, three parts of gypsum to one part of pumice. After the gypsum cast has become hard, mix eight parts of the white powdered glass with one part of the brown, wet and place in the gypsum cast, two-thirds full, remove the surplus water with a dry cloth and further dry on a hot iron plate. When hot, remove to a piece of charcoal or soldering block and fuse with a blow-pipe, being careful that only an oxidized flame comes in contact with the glass. If the gypsum model is hot first the glass will flow readily. Variation of shades may be made by adding more or less of the brown powder. Sand placed in the gypsum cast will become attached to the glass inlay, on the under side, when it is fused, furnishing a roughened surface to which the cement will adhere. After the inlay is made it is fastened in place with cement, an undercut having previously been made in the cavity.

Dr. V. H. Jackson, of New York, read a paper describing a method of

CORRECTING IRREGULARITIES AND THE APPARATUS USED.

The doctor makes use of piano wire, bent in such a way as to retain a Coffen plate without running the rubber over the teeth, and by means of a wire running across the palate, with a loop on each end, resting between the lateral incisor and first bicuspid and reaching out around the cuspid, he was able in a very short time to secure the necessary space and bring the cuspids into place. It is impossible to make intelligible a description of these methods without drawings of the models. The doctor described a method of uniting two pieces of piano wire that is novel and very useful. The ends of the wires are laid one on top of the other and wrapped with fine copper wire, or ribbon, and then the wrapped joint is covered with tinners' solder. Projections of any sort can be added easily to an appliance in this way.

Dr. G. B. Watkins, of Detroit, presented a case of regulating, where a cuspid tooth that was late in erupting had been brought down by means of a lever attached to a gold band passing around on the labial surfaces of the teeth from the second bicuspid on one side to the first molar on the other. The lever was fastened by means of a silk ligature to a screw set into the lingual surface of the cuspid.

Dr. Hall, an American dentist practicing in Shanghai, China, was introduced and invited to address the association on the status of dentistry in the eastern countries.

Dr. Hall said that the only true dental practice in those countries was among the foreign residents, and by American or European practitioners. Even in Japan little or no attention is given to conservative dentistry. The Chinese dentist will carve a rude set of teeth from bone and tie them in place, or an old woman sitting by the wayside will profess to extract worms from aching teeth, with a chop stick, thus relieving the tooth ache. There are nine American dentists practicing on the coasts of China, none in Japan and none in India. A young man of industry and talent who can not succeed in securing a remunerative practice in this country should not expect to do better in China. The monetary success does not compensate one for the deprivations connected with life in these eastern countries.

The special committee appointed to draft suitable resolutions on the death of Dr. F. H. Rehwinkel, of Chillicothe, Ohio, reported the following resolutions, which were unanimously adopted and ordered engrossed on the minutes of the meeting:

Whereas, It has pleased the Almighty Ruler of this universe to remove from this life our friend and co-worker, Dr. F. H. Rehwinkel, therefore, be it

*Resolved*, That in the death of Dr. Rehwinkel, this association and the profession at large, have sustained an irreparable loss.

*Resolved*, That as a friend and professional brother he was always most kind and faithful, and an earnest and honest worker.

*Resolved*, That to his bereaved family and friends we extend our heartfelt sympathy and condolence in their great affliction.

*Resolved*, That a copy of these resolutions be sent by the Secretary of this association to the family of the deceased brother, and that they be published in the transactions and engrossed.

Signed by Committee, { J. TAFT, W. H. MORGAN, FRANK ABBOT.

Dr. J. N. Crouse reported to the association that the International Tooth Crown Co. had served notice on him, while here in Saratoga, of suit which it had brought against him in New York, claiming damages to the amount of $60,000. He also stated that the Dental Protective Association was well heeled and able to defend him, and would do so. But he and his attorneys were of the opinion that the funds of the Protective Association should not be used to defend the officers, and that he alone would assume the expense of the defense in this suit.

A motion was made and unanimously passed appropriating $1,000, and instructing the Treasurer to honor the drafts of Dr. Crouse for the above sum, to be used by him in defending this suit.

The next place of meeting, was by ballot, decided to be Excelsior Springs, Mo., about twenty-five miles east of Kansas City.

The Executive Committee was authorized to change the date of the meeting next year to such time as would not interfere with the attendance of any one who might wish to attend the Berlin Congress.

The following officers were elected: President, Dr. M. W. Foster, of Baltimore; 1st Vice-President, Dr. A. W. Harlan, of Chicago; 2nd Vice-President, Dr. J. D. Patterson, of Kansas City; Recording Secretary, Dr. Geo. H. Cushing, of Chicago;

Corresponding Secretary, Dr. Fred. A. Levy, of Orange, N. J.; Treasurer, Dr. A. H. Fuller, of St. Louis.

Executive Committee—W. W. Walker, S. H. Guilford, S. G. Perry.

Local Committee of Arrangements—J. D. Patterson, Kansas City, Mo.; John W. Meng, Lexington, Mo.; Z. B. Hewitt, Kansas City, Mo.

Publication Committee—W. C. Barrett, J. S. Marshall, Geo. H. Cushing.

